# Supplementary material for: Development, manufacturing, and preliminary validation of a reusable half-face respirator during the COVID-19 pandemic
Source: PLoS One. 2021 Mar 17;16(3):e0247575. doi: 10.1371/journal.pone.0247575 (PMC7968700; doi:10.1371/journal.pone.0247575)
Supplement: S1 Table — (DOCX) [file pone.0247575.s001.docx]

**S1 Table. Demographics and anthropometric characteristics of participants.**

| **Participant Demographics** | **N = 8** |
| --- | --- |
| Age, mean (SD), y | 41.50 (10.14) |
| Female – no./total (%) | 2/8 (25%) |
| **Body Mass Index, categorical** no./total (%) |  |
| Under-weight (<18.5) | 0/8 (0%) |
| Normal weight (18.5-24.9) | 4/8 (50%) |
| Overweight (25-29.9) | 2/8 (25%) |
| Obese (≥30) | 2/8 (25%) |
| **Body Mass Index**, mean (SD), kg/m^2^ | 25.62 (4.01) |
| **Anthropometric** |  |
| Face Width mean (SD), mm | 136.38 (8.98) |
| Face Length mean (SD), mm | 125.25 (5.90) |
| Menton-sellion distances mean (SD), mm | 105.25 (8.55) |
| **NIOSH panel *** |  |
| 1 no./total (%) | 0/8 (0%) |
| 2 no./total (%) | 0/8 (0%) |
| 3 no./total (%) | 1/8 (12.5%) |
| 4 no./total (%) | 0/8 (0%) |
| 5 no./total (%) | 0/8 (0%) |
| 6 no./total (%) | 1/8 (12.5%) |
| 7 no./total (%) | 2/8 (25%) |
| 8 no./total (%) | 0/8 (0%) |
| 9 no./total (%) | 2/8 (25%) |
| 10 no./total (%) | 1/8 (12.5%) |
| NA no./total (%) | 1/8 (12.5%) |

* NIOSH Panel is the bivariate panel based on face-length and face-width based on the 2003 NIOSH survey anthropometric survey of respirator users across the United States. See Zhuang Z, Bradtmiller B. Head and face anthropometric survey of U.S. respirator users. J Occup Environ Hyg. 2005(2):567-576.
