# Supplementary material for: Development, manufacturing, and preliminary validation of a reusable half-face respirator during the COVID-19 pandemic
Source: PLoS One. 2021 Mar 17;16(3):e0247575. doi: 10.1371/journal.pone.0247575 (PMC7968700; doi:10.1371/journal.pone.0247575)
Supplement: S1 File — (DOCX) [file pone.0247575.s002.docx]

**3D Print Settings and Silicone casting process**

**A)** **3D Print settings**

Resolution: 0.20 mm

Infill: 20%

Nozzle: 0.4 mm

Filament: PETG

Supports: all over

**B)** **Silicone casting process**

The procedure to produce the custom silicone mask using the 3-part 3D printed mold was as follows:

1. The surfaces of the 3D printed molds were sprayed with a thin coat of Ease Release ® 200 (Mann Release Technologies Inc, Macungie, PA) and set aside to dry for approximately 10 minutes.
2. 80 mL of Dragon Skin 20 Part A and Part B was poured into two separate buckets.
3. Part B from Step 2 was poured into the bucket containing Part A. Five drops of food coloring was added in this step to obtain different colors for the mask (optional).
4. The solution was thoroughly stirred and degassed in a vacuum chamber (up to 30 inches of mercury pressure).
5. Once the solution was clear without any visible bubbles it was then poured into the mold from the top as shown in Figure 7B.
6. Procedure from Step 5 was repeated until the silicone solution slightly overflowed from the top of the mold.
7. The mold filled with the silicone was then degassed inside the vacuum chamber.
8. The silicone filled mold was removed from the vacuum chamber and silicone was again poured into the mold to ensure enough silicone was present inside to form the entire mask profile.
9. The mold was set aside to cure for at least 4 hours.
10. After curing, the screws holding the 3D printed mold were loosened and the 4 parts of the mold were separated to release the cured silicone mask.
11. Components were cleaned and disinfected in a bleach solution, then rinsed in water.
